# Supplementary material for: Exploring the effect of hypertension on retinal microvasculature using deep learning on East Asian population
Source: PLoS One. 2020 Mar 5;15(3):e0230111. doi: 10.1371/journal.pone.0230111 (PMC7058325; doi:10.1371/journal.pone.0230111)
Supplement: Attachment — Submitted filename: Review comments.docx [file pone.0230111.s001.docx]

Review comments

The article as a pilot study has highlighted some facts.

I have no further comments in the article.
