# Supplementary material for: Exploring the effect of hypertension on retinal microvasculature using deep learning on East Asian population
Source: PLoS One. 2020 Mar 5;15(3):e0230111. doi: 10.1371/journal.pone.0230111 (PMC7058325; doi:10.1371/journal.pone.0230111)
Supplement: Attachment — Submitted filename: Response to Reviewers.docx [file pone.0230111.s002.docx]

Dear academic editor and reviewer：

For the problem about the small sample size, we collected another 407 retinal photographs from patients with a diagnosis of hypertension and 405 retinal photographs from normotensive patients. There were 2012 retinal images including 1007 hypertension images and 1005 non-hypertension images in our study. The dataset size, however, was small for deep learning. So we use some strategies, such as reducing the size of the model, using dropout layers and data augmentation, to mitigate overfitting and maximize generalization. After increasing sample size, for “segmented dataset” the evaluation parameters (accuracy, specificity, precision, recall, the area under the receiver operating characteristic curve) changed little, while for “enhanced dataset” the change was comparatively large, as shown in the following table.

|  | **Enhanced dataset** | | **Segmented dataset** | |
| --- | --- | --- | --- | --- |
|  | **Before** | **After** | **Before** | **After** |
| **Accuracy** | 60.00% | 56.76% | 61.33% | 60.94% |
| **Specificity** | 61.32% | 63.80% | 53.85% | 51.54% |
| **Precision** | 59.64% | 58.97% | 59.86% | 59.27% |
| **Recall** | 56.25% | 49.93% | 68.95% | 70.48% |
| **AUC** | 0.6115 | 0.6069 | 0.6608 | 0.6506 |

Table: The performance of the model before and after increasing sample size

The possible reason was that images from “segmented dataset” contained only retinal microvasculature and the information of leading the model to hypertension decision mainly came from retinal microvasculature. But images from “enhanced dataset” contained a lot of additional information besides retinal microvasculature and the model learns representations that are not specific to hypertension. Accordingly we inferred that with increasing sample size, for “segmented dataset” the result will not be much different.

Thank you very much!

Prof. Wei He
